# European Notes; or, What I Saw in the Old World

**Published:** 1884-12

**Authors:** 


EUROPEAN NOTES, OR WHAT I SAW IN THE OLD WORLD.—By Morton Bryan Wharton, D. D., late U. S. Consul in Germany, illustrated, Atlanta, Ga., James P. Harrison & Co., Printers and Publishers, 1884.

Having just finished a most edifying perusal of the "notes of a visit to European medical centres," by William Osler, M. D., of Philadelphia at present, but recently of Montreal, in the Archives of Medicine, we took up the "European Notes" of Dr. Wharton, hoping to find some reference to the large hospitals, with their medical and surgical resources, among the other matters of interest in the old world. But not even the name of a prominent physician or surgeon appears in the list of notables encountered in London, Edinburgh, Paris, Brussels, Berlin, Leipsic, Heidelberg and Vienna, where the votaries of medical science find so much that attracts and charms them. It is true that we are told there are 2,000 physicians in London, the University of Heidelberg has 600 students, the University of Leipsic 160 professors and 3,000 students, Berlin 200 professors and 4,000 students, Vienna 150 professors and 2,500 students, and a like number of occasional attendants.

But the only commentary upon the medical departments of these magnificent centres of attraction for physicians and surgeons is in regard to the last named. It is stated that "the medical faculty is greatly distinguished, so that even here in America our aspiring young doctors, and sometimes our old ones, feel that there is no higher privilege than attending lectures in Vienna." As to most of whom he might have added, "they have ears, but they hear not," so that all is jibberish to those without a knowledge of the language.

The author of this book has evidently sought health rather in the charms of travel amidst varying scenes, than in running after the teachings of the medical craft, and though he appeared to great advantage after returning from his tour as the annual orator of one of our medical colleges in this city, his representations abroad of "American citizenship," gave a good finishing stroke to his address on this occasion. There are many points of correspondence between what he saw in the old world, and the Brazil and Brazilians of Kidder and Hatchen, gotten up under somewhat similar circumstances. The authors in each seem to have enjoyed rare opportunities of hobnobbing with royalty, and it is quite refreshing to learn that the President of the Ducal Ministry of Coburg thought that Mr. Wharton, the representative of a Republican country, who had already been received at Court, might speak and talk with her Highness when they met each other unawares. Our author seems to have taken a special interest in the fine art department of the various places visited, and the attractions of Lady Godiva crop out on two occasions; but he failed to see the most exquisite specimen of this painting in the magnificent gallery of art in Brussels, and many other graphic delineations of the old masters thus were passed unnoticed. "As we were tired viewing art collections and museums, we concluded to 'take in' this grand city by going from street to street, entering the stores and manufactories." He certainly had no occasion to go into ecstasies over the living specimens of the woman kind in Brussels, as on the occasion of the National Exposition there in 1880, amongst the large assemblages, embodying the girls from some of the French seminaries, there was not a comely maiden or damsel to be seen. On the woman question let him speak for himself, when visiting another region of the old world: "I was charmed with the appearance of the ladies in Vienna, and regard them the most beautiful of all I saw during my travels."

But no one can properly appreciate the notes of "What I saw in the old world," by a mere review of this instructive collection of observations by one who studied well the adaptation of his material to the elevation of the taste, and to the ennobling of the moral perceptions of his readers. His broad Christian exposition of the scope of Luther's work and its shortcomings, in the attainment of the great end aimed at by the Reformation, will commend itself to all who have studied the state of religious sentiment and of practical godliness throughout Germany.

The sketches of places and the word painting of society, in the various localities, are sure to interest the reader in the details of fact with which these notes abound, and no one who takes up this book will lay it down without reading, as we have done, to the last page.

